# Perspectives on barriers to implementing WHO’s five keys to safer food in resource-limited rural areas of developing countries

**DOI:** 10.3389/fnut.2025.1524580

**Published:** 2025-03-04

**Authors:** Vitowe Batch, Martina Kress, Aggrey Pemba Gama, Tinna Ng’ong’ola-Manani, Gabriella Chiutsi-Phiri, Ponyadira Leah Corner, Save Kumwenda, Chikumbusko Kaonga, Mphatso Kamndaya, Maurice Monjerezi, John F. Leslie, Limbikani Matumba

**Affiliations:** ^1^Deutsche Gesellschaft für Internationale Zusammenarbeit (GIZ) – Food Security and Nutrition Programme (FSNP)-Malawi, Lilongwe, Malawi; ^2^School of Science and Technology, Malawi University of Business and Applied Sciences, Blantyre, Malawi; ^3^Department of Food Science and Technology, Bunda College, Lilongwe University of Agriculture and Natural Resources (LUANAR), Lilongwe, Malawi; ^4^Department of Agriculture and Food Systems, Natural Resources College, Lilongwe University of Agriculture and Natural Resources (LUANAR), Lilongwe, Malawi; ^5^Department of Land and Water Systems, Natural Resources College, Lilongwe University of Agriculture and Natural Resources (LUANAR), Lilongwe, Malawi; ^6^Centre for Resilient Agri-Food Systems (CRAFS), University of Malawi, Zomba, Malawi; ^7^Department of Plant Pathology, Throckmorton Plant Sciences Center, Kansas State University, Manhattan, KS, United States

**Keywords:** cooking, food safety guidelines, food storage, hand washing, microbial contamination, mycotoxin, refrigeration, resource constrained household

## Abstract

Food contamination is a critical global health issue, with the WHO estimating millions of deaths and Disability-adjusted life years (DALYs) lost annually due to foodborne diseases, particularly in developing countries. To address this, the WHO introduced the Five Keys to Safer Food (WHO-FKSF) to promote essential food safety practices. We analyzed the limitations of the WHO-FKSF for use in developing countries. We explore contextually relevant adaptations, such as community engagement, infrastructure improvements, and innovations like durable, child-resistant tippy taps (simple, low-cost handwashing devices that dispense water when tipped) for handwashing, required to make the WHO-FKSF applicable in rural portions of developing countries. Addressing cultural norms and involving men in water-related tasks can further align food safety practices with everyday realities. We recommend providing a specific rationale for each actionable step, beyond the general reasoning provided for the five keys themselves. This additional information will improve comprehension and adherence to the practices. We also recommend revising Key 4, “Keep food at safe temperatures,” to “Store food safely” to better accommodate the lack of refrigeration and to promote low-cost food preservation methods. Adapting the WHO-FKSF to the specific needs of these communities could significantly reduce foodborne illnesses and improve public health outcomes across sub-Saharan Africa.

## 1 Introduction

Food contamination is a major global health challenge, resulting in significant morbidity and mortality, with WHO’s 2010 Foodborne Disease Burden Epidemiology Reference Group (FERG) estimates indicating millions of deaths and Disability-adjusted life years (DALYs) from foodborne diseases, comparable to malaria and tuberculosis (1, 2). This burden is especially high in Asia and Africa, where tropical climates facilitate pathogen growth particularly affecting children (3, 4). The true impact may be even greater, and updated estimates are anticipated in 2025 (5).

To combat foodborne diseases, WHO introduced the Five Keys to Safer Food (WHO-FKSF) in 2001, outlining essential food safety practices: (1) Keep clean, (2) Separate raw and cooked foods, (3) Cook thoroughly, (4) Keep food at safe temperatures, and (5) Use safe water and raw materials (6). These guidelines, translated into various languages, offer a foundational framework for improving food safety. Adherence to the WHO-FKSF depends on access to resources and the ability to integrate these practices into daily routines, which are influenced by cultural norms, social dynamics, and household responsibilities.

The WHO Five Keys to Safer Food Manual (6) provides implementation guidelines applicable primarily in urban and developed areas, where refrigeration, electricity, water and sanitation facilities are commonly available. Yet these guidelines are even more important in rural areas of developing countries, where food safety risks are significantly higher due to inadequate infrastructure, limited access to clean water, poor sanitation, and lack of refrigeration (3). Some local customs promote unhygienic activities, e.g., limited handwashing, and common utensils and surfaces for kitchen and non-kitchen purposes, that need non-scientific efforts to change (7). The reliance on traditional food preparation methods can further increase the risk of contamination. Unlike urban settings, where modern facilities and regulations may mitigate foodborne threats, rural communities experience a disproportionately higher burden of foodborne diseases (8, 9). Adhering to the WHO-FKSF and identifying functional adaptations for rural developing situations can help mitigate these risks by promoting hygiene, safe food handling, and proper storage, thereby reducing illness and improving public health.

WHO-FKSF guidelines are scientifically robust, but implementing them in rural, resource-limited settings present unique and complex challenges. These challenges are influenced by a combination of cultural norms, societal structures, and resource constraints that affect food safety practices and attitudes toward them. This perspective explores known barriers to adherence to the Five Keys in resource-limited communities in developing countries. As a result, we propose adapting WHO’s food safety strategies to better align with the everyday realities and constraints of rural life in these regions—Five Keys to Safer Food in Developing Countries (FKSF-DC).

## 2 Challenges to implementing WHO’s five keys to safer food in developing countries

### 2.1 Key 1: Keep clean

In many households, limited access to running water forces communities to rely on alternatives like tippy taps (10) for handwashing. These simple devices, typically consisting of containers that dispense water when tipped via a foot lever, are frequently damaged by children and livestock, making them difficult to maintain. A recent study found that only 10.5% of households had functional tippy taps, all located near toilets, with none placed near kitchens where food is prepared (7). The scarcity and specific placement of these stations likely increase their novelty, making them attractive as playthings for children, leading to frequent misuse and damage. The lack of adequate household water exacerbates these problems. Many households rely on water drawn from distant sources that is carried home in jugs or pails carried by women on their heads. Bringing water is time-consuming and is only one of many responsibilities that women bear, e.g., hygiene, farming, child care, etc. (11). Thus, clean household water is at a premium, very limited in quantity and its use restricted to essential activities with drinking and cooking usually prioritized over hygiene practices such as handwashing, and cleaning of utensils and work surfaces (12).

Soap also is perceived as a luxury due to competing household priorities (13), and its cost often renders it inaccessible in many communities. From a recent focus group, “We lack soap for bathing, so how can we afford the luxury of frequent hand washing with soap?” (Batch et al., unpublished). Inadequate knowledge of the health benefits of handwashing and social norms hinder compliance even further, as deeply rooted beliefs and behaviors can undermine the adoption of consistent hand hygiene practices. For example, from a focus group participant, “If you insist on proper hand washing in public such as at a funeral, church, wedding or in the company of your in-laws, they will ridicule you and say you are selfish and pompous” (Batch et al., unpublished). The improper use and handling of kitchen cloths also compounds hygiene risks, as cloths are often reused without adequate washing or drying, creating a breeding ground for pathogens. The necessity of safeguarding livestock from theft leads many households to keep animals within close proximity, further complicating food safety practices (14).

### 2.2 Key 2: Separate raw and cooked foods

Economic constraints prevent families from purchasing enough utensils to separate raw and cooked foods, a challenge worsened by the scarcity of potable water and soap, which renders washing between uses ineffective if it occurs at all (15). Home-slaughtered livestock, especially chickens, are desirable, but less common, and the same utensils are frequently used for both raw and cooked meats or other dishes leading to frequent cross-contamination. Furthermore, cultural priorities often place the importance of dish and utensil purchases below other household expenses such as food, school fees, and medical costs, and duplicate sets of bowls, plates and serving utensils are often viewed as a luxury that is unaffordable and perhaps even unnecessary.

### 2.3 Key 3: Cook food thoroughly

Thermometers are unavailable or are too expensive, which leads to reliance on visual cues such as clear juices or browning, which may not suffice to ensure safety. Limited access to affordable fuel restricts cooking time, particularly for large cuts of meat. Many households lack electricity, much less devices such as microwaves. Using only wood or charcoal fires makes it difficult to reheat food evenly and safely (16).

### 2.4 Key 4: Keeping food at safe temperatures

Most households in developing countries lack refrigerators and ice boxes for cooling, forcing reliance on unsafe food preservation methods. While solar-powered refrigeration can serve as an alternative, its high cost makes it too expensive for many (17). Further, the cold chain is not always available during distribution and retail sale of fresh and highly perishable foods in informal markets which are the main sources of these foods for most households. Meat usually is boiled, although it may also be fried or grilled over an open fire. Cooked food often is kept warm by simmering, which commonly requires wood or charcoal to keep a fire going and may not evenly distribute the heat across the pot or pan. In general, other than dried grains and similar products, food, whether raw or cooked, is not preserved and must be consumed the day it is acquired and processed.

### 2.5 Key 5: Using safe water and raw materials

**Figure 1:**
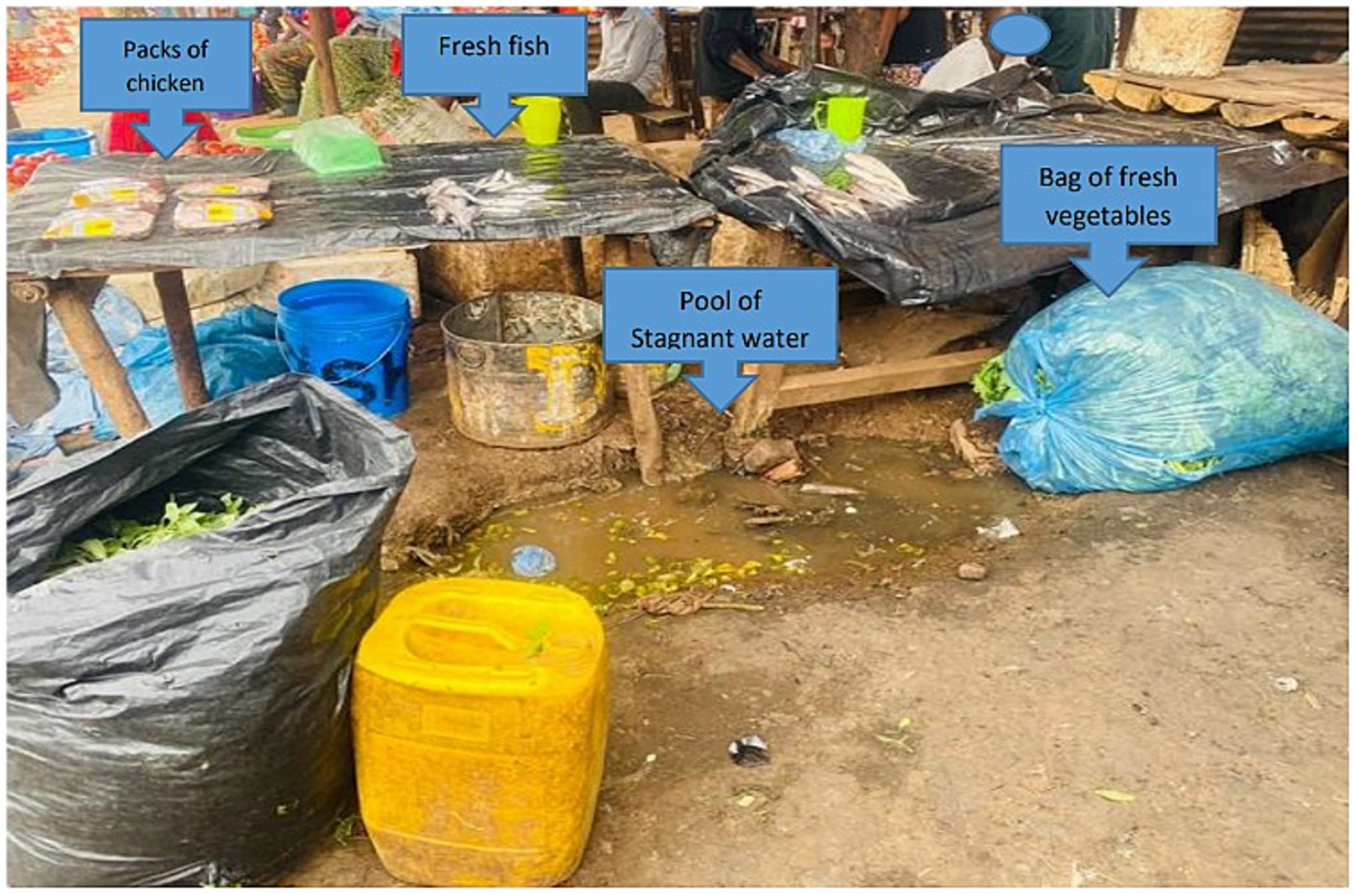
Typical unhygienic conditions in a local market in Lilongwe (Photograph by Tinna Manani).

Many households in developing countries must fetch water from distant sources, a task predominantly carried out by women. Due to their numerous other responsibilities, such as childcare, cooking, and farming, women often cannot fetch sufficient water to meet all household needs, such as cooking, hygiene and sanitation. Water treatment chemicals either are unavailable or too expensive. Boiling water may change its taste, requires more firewood, and increases expense and deforestation. In many locations, it is all but impossible to obtain safe raw materials as hygiene is neither prioritized nor viewed as important. Local marketplaces commonly are unkempt at best (Figure 1), yet consumers continue to patronize them without questioning the safety of the products they purchase. Foods in these markets are normally not refrigerated. They commonly are open to the air and may be stored on the ground where they can be easily mixed with soil, animal dung, food waste and other debris and acquire microbial contaminants from the air or other sources.

## 3 Discussion

Implementation of stringent food safety guidelines in developing countries is critical. The high ambient temperatures in many of these countries are conducive to the proliferation of foodborne pathogens, significantly elevating the risk of foodborne illnesses. Paradoxically, despite the heightened need, the implementation of WHO-FKSF faces considerable challenges in these regions. These obstacles are rooted in a combination of cultural norms and infrastructural, educational, and resource limitations, which hinder effective adherence to these essential food safety measures.

The WHO-FKSF are premised on the assumption that communities have relatively easy access to essential resources such as clean running water, soap, refrigeration, and proper cooking utensils, each of which is critical for preventing food contamination and ensuring food safety (3, 4). Unfortunately, in many rural areas of developing countries, these fundamental resources are lacking, or are not present at all. Most rural residents lack access to safe drinking water, and may have limited access to water for other purposes. Only a small proportion of rural households are connected to electricity, which makes refrigeration all but unobtainable. These shortages, compounded by inadequate sanitation facilities and insufficient cooking tools, undermine the practical implementation of the WHO-FKSF guidelines. The gap between the premise of available resources and the reality on the ground highlights the need for food safety strategies that are adapted to these local constraints.

Cultural norms and societal structures also present additional barriers. In many rural areas, deeply entrenched gender roles dictate that women are solely responsible for water collection, cooking, and other household tasks, giving them the primary responsibility for food safety. These roles limit their time and capacity to consistently follow the WHO-FKSF guidelines. Involving men and boys in water-related tasks can alleviate some of these burdens. For example, in India when men participate in water-fetching, they often use technology or transport methods that increases efficiency, reduces physical strain, and enables more sustainable approaches to meeting household water needs (18).

The omission of traditional preservation methods, e.g., drying, smoking, and fermenting, and the lack of guidance on microbial and mycotoxin contamination control, reveal a bias in the guidelines for resource-advantaged settings. The omission of these methods means that subsistence communities that rely on these methods lack guidance for contamination control, as highlighted under key number 5 (19). For example, integrating procedures specifically focused on mycotoxin control, such as those proposed by Matumba et al. (20), could increase the applicability of the WHO-FKSF in these settings. Such changes also align with the recommendations of Langsrud et al. (21), who advocate a revision of the WHO-FKSF that adopts a multidisciplinary, consumer-centered approach. Effective food safety communication addresses consumer motivations, cultural contexts, and practical challenges, including resource limitations and local practices. Such revisions would make the WHO-FKSF more adaptable, relevant, and applicable to food safety outcomes across diverse environments.

To increase the applicability of the WHO-FKSF in rural, resource-limited settings, these guidelines need to address local cultural practices, resource constraints, and societal structures. Engaging community leaders in promoting context-specific adaptations can make these hygiene practices more relevant. Child-resistant, durable designs for tippy taps can minimize misuse and prolong functionality, supporting consistent hand washing practices. Encouraging men’s involvement in water-related tasks could distribute some household tasks more equitably and promote innovative water solutions. Integrating affordable, well-established food preservation alternatives, e.g., fermentation and drying, can reduce the demand for refrigeration, while specific strategies for mycotoxin management and livestock containment can mitigate contamination risks in subsistence farming communities. Further, consumers should be taught to consider the hygienic conditions in open markets and to select food only from hygienic stalls.

We propose modifying the WHO-FKSF guidelines to better align with the realities of resource-limited settings. In particular, we recommend revising Key 4, “Keep food at safe temperatures,” to “Store food safely” (Table 1). This change expands the guideline to include alternatives to refrigeration, that are more readily implemented in resource-constrained developing countries, where refrigeration infrastructure is limited or non-existent. Additionally, we suggest providing a clear, evidence-based rationale for each overarching key and its specific actions. For example, as part of the “Keep clean” key (Key no. 1), the rationale for using running water during handwashing should clearly explain why washing in running water is more effective than is washing in stagnant water. By offering clear, scientifically supported explanations for each key action, the guidelines will be easier to understand, and more likely to be adopted, as individuals will more easily see the practical benefits of these food safety practices.

**Table 1:** Proposed modification of the WHO five keys to safer food’s fourth key, from “Keep food at safe temperatures” to “Store food safely” for regions with limited refrigeration.

Key 4: Store food safely		
What	Why	How to actualize
Refrigeration and freezing^1^		
Refrigerate promptly all cooked and perishable foods at temperatures <5°C^1^.	Low temperatures slow microbial growth, preserving the food’s safety and extending its shelf life.	Store perishable foods in a refrigerator as soon as possible after purchase or cooking. Use a refrigerator thermometer to confirm that the temperature stays below 5°C.
Limit storage time, even in a refrigerator. Cooked food should be consumed or preserved within 3 days^1^.	Some microbes grow slowly even when food is refrigerated. Limiting storage time reduces spoilage risk.	Label containers with the date of storage and set reminders to consume, preserve or dispose of cooked food within 3 days.
Do not thaw frozen food at room temperature. Always thaw food in a refrigerator or another cool place^1^.	Thawing food at room temperature encourages bacterial growth on the food surface. Thawing in the refrigerator maintains a safe temperature throughout the process.	Plan ahead to allow enough time for food to thaw in a refrigerator. Alternatively, use a cold-water bath or microwave to speed up the process while keeping the temperature safe. Food thawed in a microwave oven should be cooked promptly.
Heated food		
Keep cooked food piping hot (> 60°C) if storing for immediate consumption^2^^,^^3^.	Food at >60°C does not support microbial growth in the “danger zone” between 5°C and 60°C.	Use a food thermometer to ensure hot food stays above 60°C. Cover food and keep it in an insulated container if it needs to stay hot for an extended period of time.
Storage at ambient temperature		
Obtain fresh food daily and prepare only what is needed.	Without refrigeration, fresh food has a short shelf life.	Purchase fresh food in small quantities, plan meals carefully, and prepare portions that match immediate consumption needs. Avoid leftovers when refrigeration is not available.
Do not leave cooked food exposed for long periods of time (consume within 2 h at room temperature)^4^^,^^5^.	Microorganisms multiply rapidly at ambient temperatures. Eating within 2 h reduces microbial growth risk.	Set timers to track how long food is exposed. Cover food when it is left out and refrigerate leftovers promptly.
Dehydrate/dry foods such as vegetables, fruits, fish and meats to reduce moisture.^4,5^	Removing water lowers food’s moisture and inhibits the growth of bacteria, molds, and yeasts.	Use solar dryers, electric dehydrators, or simply dry food in the sun. Ensure that food is dried thoroughly and stored in airtight containers to prevent moisture reabsorption.
Salt meat, fish, and vegetables to sequester moisture and inhibit microbial growth.^4,5^	High levels of salt create environments that draw moisture out of microbes and stops them from growing.	Rub salt evenly on the surface of the food or submerge the food in brine. Store treated food in a cool, dry place to increase effectiveness. Note: This method is not recommended for individuals with high blood pressure for whom excessive sodium intake may pose a health risk.
Smoke meats and fish to preserve them.^4,5^	Smoking introduces anti-microbial compounds while simultaneously drying the food at a temperature high enough to restrict or prevent microbial growth.	Smoke food at low temperatures using wood chips or charcoal in a smoker. Ensure the food is cured or brined beforehand. Store in a cool, dry place after smoking.
Ferment foods like vegetables (e.g., pickles or kimchi) and dairy (e.g., yogurt).^4,5^	Fermentation usually lowers pH and may create an atmosphere lacking O_2_, that restricts or prevents harmful microbial growth.	Use appropriate starter cultures or naturally occurring microbes. Follow tested recipes to ensure the right balance of ingredients and storage conditions for fermentation.
Marinade food in acidic solutions, e.g., vinegar.^4,5^	Acidic solutions inhibit the growth of spoilage microorganisms.	Prepare acidic marinades with vinegar or citrus juice and submerge the food for sufficient time. Keep the food covered and, when possible, refrigerated during marinating.
Can food in jars^4^	Canning removes air required for microbial growth on both refrigerated and dried foods.	Sterilize jars for canning by placing them in boiling water before filling them. Ensure a tight seal after processing. Store sealed foods according to their type (refrigerated or shelf-stable).

Rationale: Microorganisms can multiply quickly if food is stored at room temperature or retains too much moisture. Preservation techniques focus on lowering the amount of moisture in the food (water activity) and/or altering temperature, to slow or stop the growth of harmful microorganisms.

1 United States Department of Agriculture (28).

2 United States Department of Agriculture (29).

3 United States Department of Agriculture (30).

4 Norris (31).

5 Gardeners and Farmers of Centre Terre Vivante (32).

In addition to individual responsibilities, the government must play a crucial role (22) by providing educational resources and in creating an enabling environment for compliance with the WHO-FKSK guidelines. In this role, four key areas must be addressed: (i) access to safe water, (ii) reliable electricity infrastructure, (iii) public education, and (iv) the affordability of hygiene essentials (23–27). Safe water is fundamental for cooking, cleaning, and handwashing, preventing contamination and foodborne illnesses. A stable electricity supply supports refrigeration, reducing spoilage and extending the shelf life of perishable foods. Public education initiatives should enhance awareness of proper hygiene practices and safe food handling. Finally, ensuring that hygiene products, e.g., hand and dish soap, and water dispensers, remain affordable will encourage their widespread, consistent use and help minimize microbial contamination during food preparation. By prioritizing these four areas, the government can strengthen public health and food security, and foster a safer and healthier society.

## 4 Conclusion

In rural portions of developing countries the stark reality is that unless food safety guidelines are adapted to local conditions, the most vulnerable communities may be left behind. The WHO-FKSF must evolve beyond its current framework to incorporate cultural understanding and resource-adaptive strategies to create meaningful change. As a first step in this evolution, we suggest that the WHO Five Keys to Safer Food (WHO-FKSF)—(1) Keep clean, (2) Separate raw and cooked foods, (3) Cook thoroughly, (4) Keep food at safe temperatures, and (5) Use safe water and raw materials—be changed to FKSF-DC—(1) Keep clean, (2) Separate raw and cooked foods, (3) Cook thoroughly, (4) ***Store food safely***, and (5) Use safe water and raw materials. Further, guidelines should be developed to address particular regions’ socio-economic realities. By making this change, these guidelines will impact situations in developing countries that are in greatest need of improvement in food safety.

## Data Availability

The raw data supporting the conclusions of this article will be made available by the authors, without undue reservation.
